# Supplementary material for: Conditional chemoconnectomics (cCCTomics) as a strategy for efficient and conditional targeting of chemical transmission
Source: eLife. 2024 Apr 30;12:RP91927. doi: 10.7554/eLife.91927 (PMC11060718; doi:10.7554/eLife.91927)
Supplement: Figure 3—source data 3. [file elife-91927-fig3-data3.zip › Figure 3 source data3 part 2.zip!/Figure 3-Source data 3-part2/1063-1-CRISPResso_Report_az97gqtx.zip!/CRISPResso_on_1063_3/9.Alleles_frequency_table_around_sgRNA_ggatgtttccggccaccgaa.pdf]

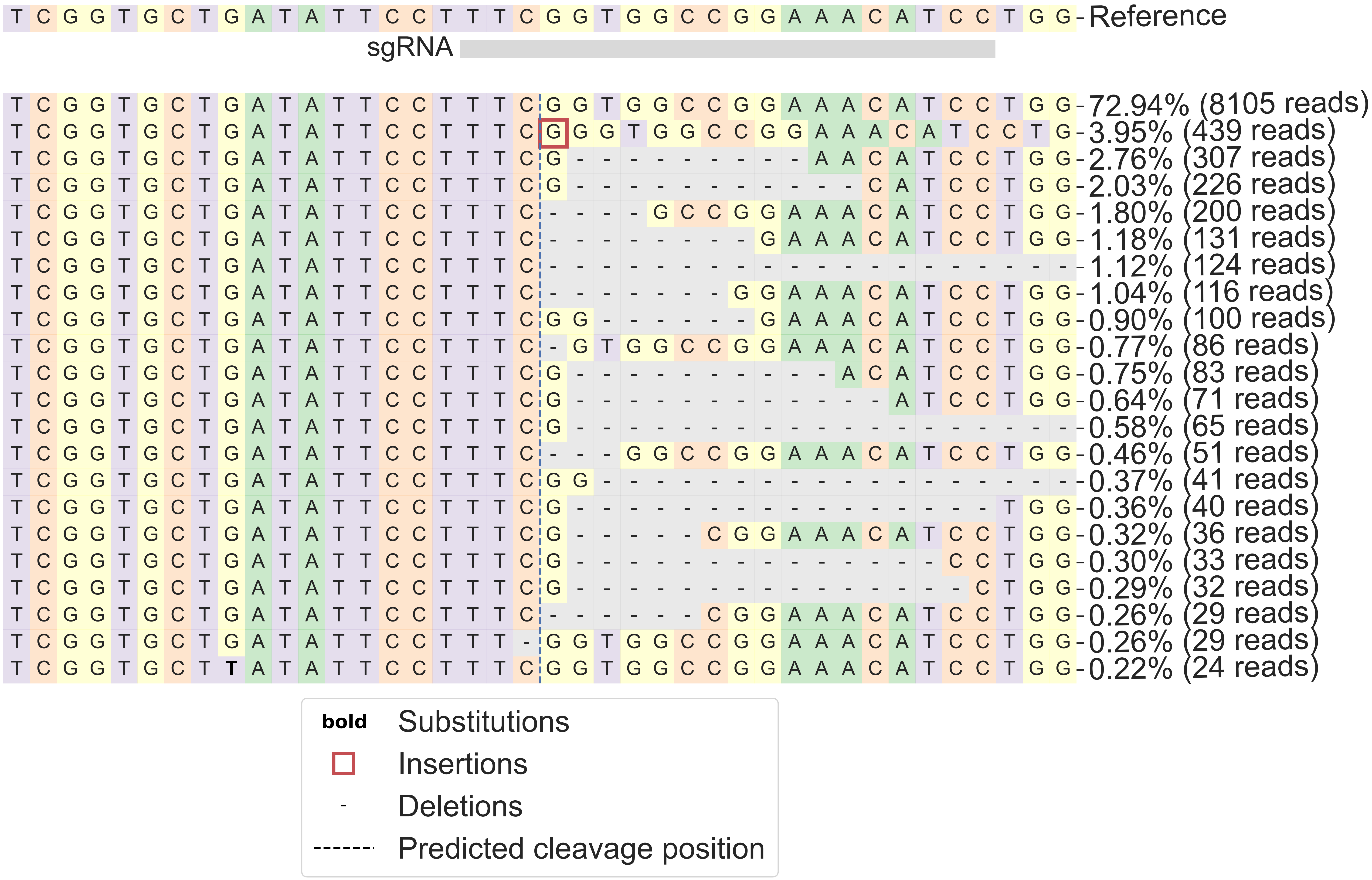
TCGGTGCTGATATTCCTTTCGGTGGCCGGAAACATCCTGG - Reference
sgRNA
TCGGTGCTGATATTCCTTTCGGTGGCCGGAAACATCCTGG - 72.94% (8105 reads)
TCGGTGCTGATATTCCTTTCGGGTGGCCGGAAACATCCTG - 3.95% (439 reads)
TCGGTGCTGATATTCCTTTCG---------AACATCCTGG - 2.76% (307 reads)
TCGGTGCTGATATTCCTTTCG-----------CATCCTGG - 2.03% (226 reads)
TCGGTGCTGATATTCCTTTC----GCCGGAAACATCCTGG - 1.80% (200 reads)
TCGGTGCTGATATTCCTTTC--------GAAACATCCTGG - 1.18% (131 reads)
TCGGTGCTGATATTCCTTTC-------------------- - 1.12% (124 reads)
TCGGTGCTGATATTCCTTTC-------GGAAACATCCTGG - 1.04% (116 reads)
TCGGTGCTGATATTCCTTTCGG------GAAACATCCTGG - 0.90% (100 reads)
TCGGTGCTGATATTCCTTTC-GTGGCCGGAAACATCCTGG - 0.77% (86 reads)
TCGGTGCTGATATTCCTTTCG----------ACATCCTGG - 0.75% (83 reads)
TCGGTGCTGATATTCCTTTCG------------ATCCTGG - 0.64% (71 reads)
TCGGTGCTGATATTCCTTTCG------------------- - 0.58% (65 reads)
TCGGTGCTGATATTCCTTTC---GGCCGGAAACATCCTGG - 0.46% (51 reads)
TCGGTGCTGATATTCCTTTCGG------------------ - 0.37% (41 reads)
TCGGTGCTGATATTCCTTTCG----------------TGG - 0.36% (40 reads)
TCGGTGCTGATATTCCTTTCG-----CGGAAACATCCTGG - 0.32% (36 reads)
TCGGTGCTGATATTCCTTTCG--------------CCTGG - 0.30% (33 reads)
TCGGTGCTGATATTCCTTTCG---------------CTGG - 0.29% (32 reads)
TCGGTGCTGATATTCCTTTC------CGGAAACATCCTGG - 0.26% (29 reads)
TCGGTGCTGATATTCCTTT-GGTGGCCGGAAACATCCTGG - 0.26% (29 reads)
TCGGTGCT**T**ATATTCCTTTCGGTGGCCGGAAACATCCTGG - 0.22% (24 reads)
bold Substitutions
Insertions
- Deletions
------ Predicted cleavage position
